# Fear learning induces structural and functional plasticity at GABAergic synapses in the basolateral amygdala

**DOI:** 10.1186/1471-2210-11-S2-A42

**Published:** 2011-09-05

**Authors:** Yu Kasugai, Elisabeth Vogel, Markus Hauschild, Ramon O Tasan, Yvan Peterschmitt, Andreas Lüthi, Ryuichi Shigemoto, Werner Sieghart, Nicolas Singewald, Günther Sperk, Francesco Ferraguti

**Affiliations:** 1 Department of Pharmacology, Innsbruck Medical University, 6020 Innsbruck, Austria; 2 Friedrich Miescher Institute for Biomedical Research, 4058 Basel, Switzerland; 3 Department of Pharmacology and Toxicology, Institute of Pharmacy, and Center for Molecular Biosciences Innsbruck (CMBI), University of Innsbruck, 6020 Innsbruck, Austria; 4 Division of Cerebral Structure, National Institute for Physiological Sciences, Okazaki 444-8787, Japan; 5 Department of Biochemistry and Molecular Biology, Center for Brain Research, Medical University of Vienna, 1090 Vienna, Austria

## Background

Previous work has suggested that alterations in GABAergic function within the amygdala underlie fear learning. In particular, it has been shown that Pavlovian fear conditioning induces a downregulation of benzodiazepine binding sites as well as transcripts for gephyrin and some GABA_A_ receptor subunits in the basal nucleus of the amygdala (BA), which were restored to control levels after fear extinction.

## Methods

We have undertaken a combined anatomical and physiological approach to examine whether these alterations distinctively involve GABA_A_ receptors in synaptic or extrasynaptic areas. Specifically, we analyzed – in the BA of mice that underwent fear conditioning as well as extinction – miniature inhibitory postsynaptic currents (mIPSCs), mRNA levels by *in situ* hybridization, and the density for the GABA_A_ γ2 subunit by means of the freeze-fracture replica immunolabelling technique (SDS-FRL). SDS-FRL also allowed to precisely measure the size of GABAergic synapses.

## Results

A significant decrease in labelling density for the GABA_A_ γ2 subunit could be detected in the synaptic area in fear-conditioned mice as compared to the control group and mice that had undergone extinction (p < 0.01; Kruskal-Wallis and Dunn’s multiple comparison tests). Conversely, GABA_A_ γ_2_ extrasynaptic density was lower in the extinction group when compared to both the control and fear-conditioned mice (p < 0.005). The average size of GABAergic synapses in control mice was 0.034 ± 0.001 µm^2^ (n = 227 full synapses from 3 animals; CV = 0.62). Fear-conditioned animals showed a significantly (p < 0.01) larger average synaptic size (0.040 ± 0.001 µm^2^; n = 249; CV = 0.59), whereas in fear extinction mice it was similar to controls (0.031 ± 0.001 µm^2^; n = 290; CV = 0.59). Alterations in synapse size upon fear conditioning and extinction were associated with functional changes. In neurons recorded from acute slices obtained from fear-conditioned animals mIPSCs were larger (increased charge transfer/mIPSC) compared to recordings obtained from slices of control mice and animals subjected to extinction training. *In situ* hybridization analysis of the mRNA content for GABA_A_ γ2 subunits revealed highly similar levels among the 3 groups in the BA (p = 31, one-way ANOVA) and central nucleus (p = 41).

## Conclusions

Our results indicate that, in the BA, fear conditioning produces a reversible enlargement of GABAergic synapses and an increase in mIPSC charge transfer with no change in the overall number of synaptic GABA_A_ receptors.

